# Use of Contrast-Enhanced Ultrasound of the Testes after Non-Surgical Sterilization of Male Dogs with CaCl_2_ in Alcohol

**DOI:** 10.3390/ani12050577

**Published:** 2022-02-25

**Authors:** Vincenzo Cicirelli, Francesco Macrì, Simona Di Pietro, Raffaella Leoci, Giovanni Michele Lacalandra, Giulio Guido Aiudi

**Affiliations:** 1 Department of Veterinary Medicine, University of Bari Aldo Moro, S.P. 62 per Casamassima, Km 3, Valenzano, 70010 Bari, Italy; vin.cicirelli@libero.it (V.C.); leocivet@yahoo.it (R.L.); giovannimichele.lacalandra@uniba.it (G.M.L.); g.aiudi@veterinaria.uniba.it (G.G.A.); 2 Department of Veterinary Science, University of Messina, Viale Palatucci, 98168 Messina, Italy; francesco.macri@unime.it

**Keywords:** calcium chloride, chemical castration, contrast-enhanced ultrasound, dog

## Abstract

**Simple Summary:**

The uncontrolled stray dog population presents a serious threat to public health due to the increased risk of transmitting zoonotic diseases and inflicting human injury. There is a growing interest in non-surgical chemical sterilization via intratesticular injection of chemical neutering agents such as calcium chloride, which causes sterility due to azoospermia. In this study, we monitored immediate vascular alterations in dogs from chemical sterilization, using contrast-enhanced ultrasound (CEUS) before and after intratesticular inoculation with calcium chloride. The CEUS is an imaging technique used to obtain a quali-quantitative assessment of tissue perfusion by intravenously injecting gas-filled microbubble contrast agent. The CEUS evaluation was performed in canine testes before and after intratesticular inoculation with calcium chloride. Our results showed that the hardening agent caused a drastic reduction of the intratesticular blood flow, which was reflected by sonographic findings. CEUS can help clinicians define immediate testicular vascular alterations achieved by chemical castration. Nevertheless, more studies are required to apply this methodology to more subjects in a broader weight range among stray dogs.

**Abstract:**

Sterilization by intratesticular injection of chemical agents is a non-surgical alternative to neutering male companion animals. We used contrast-enhanced ultrasound (CEUS) to monitor vascular alterations to testes immediately after the intratesticular injection of CaCL_2_ in alcohol. We evaluated the CEUS features of normal and damaged testes in 20 dogs after the intratesticular injection of CaCl_2_. The CEUS evaluation was performed at the site of the chemical agent inoculation. In treated testes, qualitative CEUS showed a lower intensity enhancement of the parenchyma than pre-treatment normal testes with a predominantly anechoic pattern and only a few hyperechoic vascular focal spots. Quantitative CEUS showed significantly lower values of time-intensity curve (TIC) parameters, including signal intensity (Peak: 4.72 ± 2.1), regional blood volume (RBV: 134.3 ± 63.7), and regional blood flow (RBF: 4.36 ± 2.18) than normal testes (*p* < 0.001). Sonographic findings from CEUS showed hypovascularization of the canine testicular parenchyma caused by the hardening agent. This diagnostic technique helps clinicians define testicular vascular alterations achieved by chemical castration more efficiently. Nevertheless, more studies are required to apply this methodology to more subjects with a broader weight range and stray dogs.

## 1. Introduction

In many countries, the uncontrolled stray dog population presents a serious threat to public health due to the increased risk of transmitting zoonotic diseases and inflicting human injury from bites, fractures, and road accidents [1,2].

Orchiectomy is a commonly accepted surgical method for managing the overpopulation of stray dogs [3] that achieves physical and behavioral benefits in neutered male animals [4]. Despite the technical simplicity and effectiveness of the procedure, an orchiectomy is a surgical approach that requires anesthesia, surgical equipment, recovery time, and adequate follow-up. Furthermore, the surgical procedure can be expensive to perform on a large scale and carries the risks of infection or bleeding [5,6,7].

Many studies have been conducted to simplify the sterilization process in male dogs, reducing the cost and surgical requirements associated with orchiectomy and allowing for the large-scale sterilization of male dogs [1,5,8]. Additionally, a non-surgical alternative may also appeal to dog owners who are averse to surgical castration. There is a growing interest in non-surgical chemical sterilization causing infertility and azoospermia in males by injecting a predetermined amount of chemical neutering agent into the testis, epididymis, or vas deferens [5]. This affordable method shows many advantages compared with the surgical approach, such as safety, apparent reduction of pain and stress, and eliminating surgical sequelae. Moreover, it offers immense benefits and allows animal welfare organizations, public health programs, and governments to reduce dog overpopulation even with limited resources [2,5].

In previous years, a variety of chemical agents for castrating male dogs have been used [8,9,10,11,12,13,14]. Calcium chloride (CaCl_2_) in saline, lidocaine, or alcohol-based solutions that have been effectively used as sclerosing agents in different species of male animals, including dogs [3,5,15]. We recently confirmed the outcome of this treatment in dogs by reporting long-term azoospermia in treated animals with a significant reduction in blood testosterone and aggressive and/or sexual behavior, without side effects [16]. Since the sclerosing substance severely altered the testicular parenchyma, leading to vascular injury and reduced blood perfusion, we decided to evaluate whether testicular damage appeared immediately after the CaCL_2_ injection in canine testes in our study. We hypothesized that quantifying testicular perfusion from contrast-enhanced ultrasound (CEUS) may be valuable for understanding the immediate vascular alterations to testes.

CEUS is an imaging technique used to obtain a reliable quantification of tissue perfusion based on a region of interest (ROI) analysis. It is performed by injecting gas-filled microbubbles with a diameter of less than 6–8 μm and stabilized by an outer shell into the bloodstream. These microbubbles enhance microcirculation and provide a quali-quantitative assessment of perfusion changes in an organ or tissue in real-time [17]. This diagnostic technique represents a quick, effective, and low-cost way of demonstrating the action of sclerotic solutions on testicular vessels.

Therefore, our study aimed to monitor immediate vascular alterations in the testicular parenchyma using CEUS after injecting CaCL_2_ in alcohol for non-surgical castration in dogs.

## 2. Materials and Methods

### 2.1. Animals

The present study was performed at the Veterinary Teaching Hospital of the University of Bari Aldo Moro from June to December 2016. 

Twenty client-owned dogs meeting the following requirements were recruited for our study: age 3–6 years, weight 12–30 kg, no known previous pathologies, and low anesthetic risk (American Society of Anesthesiology class 1). The present study was approved by the veterinary board of the Ethical Committee of the Department of Emergency and Organ Transplantation at the University of Bari Aldo Moro, Italy (DETO 11 April 2016). All treatments, housing, and animal care reported in this study were conducted in accordance with the EU Directive 2010/63/EU for the protection of animals used for scientific purposes. All dog owners provided their informed consent in writing. 

The dogs represented a sub-set of the population used in previous studies with sclerotizing agents.

The dogs were routinely dewormed and vaccinated. Each animal underwent a complete physical examination [18] and laboratory exams, including blood count (hematocrit, erythrocyte, leukocyte, and platelet counts, hemoglobin and mean corpuscular hemoglobin concentration), and hematochemical profile (urea, creatinine, total protein, albumin, glutamate pyruvate transferase, gamma-glutamyl transferase, and aspartate aminotransferase). 

To assess the reproductive capability of the enrolled dogs, a physical and ultrasonographic examination of the reproductive tract and an evaluation of semen quality were performed before the start of the study [19]. The penis and prepuce, spermatic cord and scrotum, epididymis and testes were examined for any evidence of inflammation or abnormality and pain assessment. The prostate was palpated to evaluate its size, consistency, symmetry, and texture. B-mode ultrasonography was also performed for the testes, epididymis, and prostate. Semen samples were collected by digital manipulation [20] and analyzed by computer-assisted sperm analysis (CASA) within 15 min to evaluate the concentration and total motility of spermatozoa [21]. We confirmed our results using optical microscopy evaluation and compared them to previous reports [22]. The enrolled dogs had a spermatozoa count of >300 million in the ejaculate and total motility >70%, within the normal range for canine species [7]. An assay for serum testosterone concentration was also performed, showing values within the normal range for dogs, as previously reported [3]. 

### 2.2. Intratesticular Injection Protocol

The experimental solution was prepared with 20 g of CaCl_2_ powder (Merck KGaA, Darmstadt, Germania), added to 100 mL of 95% ethanol (Baker Analysed ACS, JT Baker, Fisher Scientific, Rodano (MI), Italy), mixed and divided into Falcon tubes (Fisher Scientific, Rodano (MI), Italy), and sterilized by autoclaving, as previously reported [3]. The experimental solution was used at room temperature. The dose administered was determined based on the testicular size, as measured with a caliper. The testicular length, width, and height were measured in millimeters after stretching the scrotal skin over the testes, as previously reported [23]. Furthermore, the ultrasonographic measurement of testicular dimensions was performed using longitudinal and transverse scan planes. Dogs with testicular diameters of 15–18 mm were injected with 0.5 mL; diameters of 19–22 mm, 0.8 mL, and diameters ≥23 mm, 1.0 mL. 

All dogs were sedated using tiletamine chlorhydrate and zolazepam chlorhydrate (Zoletil 100, Virbac^®^, Milan, Italy) at a dosage of 6.6 mg/kg intramuscularly (IM). A fentanyl bolus (Fentanest; Pfizer Italia Srl, Latina, Italy) of 0.002 mg/kg was administered intravenously (IV) for analgesia.

Each dog was placed in a lateral recumbent position. After an accurate clipping and disinfection of the testicular skin, the sclerosing agent was injected using a 23G needle (Fisher Scientific, Rodano (MI), Italy) inserted from the caudal pole of each testis and directed towards the opposite pole. The needle was quickly retracted after carefully depositing the solution, paying attention to avoid reflux of the agent. Then, the conventional and CEUS ultrasonography of the testes was performed.

### 2.3. Ultrasound Examination Protocols

The ultrasound studies were performed before and immediately after CaCL_2_ intratesticular injection by the same investigator (F.M.) using a system equipped with contrast-tuned imaging technology (MyLab™ ClassC, ESAOTE s.p.a., Genova, Italy) and a multi-frequency 9-2 MHz linear probe (L4-15 apple probe VET, ESAOTE s.p.a., Genova, Italy). Transverse and longitudinal planes were used to assess the testes. Real-time, two-dimensional greyscale ultrasound images of testicular parenchyma followed by color doppler ultrasound () were used to detect the presence or absence of vascular flow. 

During CEUS, the mechanical index (MI) was set between 0.08 and 0.09, and a single focal zone was placed under the testicles. A sulphur hexafluoride microbubble signal enhancer (SonoVue, Bracco Imaging, Milan, Italy) was prepared according to the manufacturer’s recommendations and used as a contrast agent. Each 25 mg vial of freeze-dried powder was reconstituted with 5 mL of 0.9% sodium chloride. The vials were then vigorously shaken for 20 s. The contrast agent was used at room temperature. Once an intravenous catheter of 18–20 G was inserted in the cephalic vein, an aliquot (0.03–0.04 mL/kg body weight) of the contrast medium was rapidly injected using a three-way valve. After the contrast injection, a 5 mL saline flush was immediately performed. Two bolus injections of contrast agent were administered 8–10 min apart in each dog. The first bolus helped evaluate the pattern of the normal testicular parenchyma. The second bolus was injected a few minutes after the intratesticular administration of the CaCl_2_ solution to evaluate any vascular damage. The CEUS evaluation was performed at the site of the chemical agent inoculation. The timer and video clips were activated simultaneously with the contrast injection. 

After the procedure, all dogs were hospitalized and monitored until fully awake for their rectal temperature, heart, and respiratory rates. A digital thermometer (Adtemp™ 422, New York, NY, USA) was used to measure temperature. Heart rate was assessed using a stethoscope, and the cardiac rhythm by electrocardiography using lead II. The respiratory rate was calculated from thorax excursions. The dogs’ pain was also evaluated 2, 12, and 24 h after the procedure, using the short form of the Glasgow Composite Pain Scale (GCPS-SF), as previously reported [24]. Analgesic and anti-inflammatory treatments were performed using tramadol (Altadol 50 mg/mL, Formevet s.r.l., Milano, Italy) at a dosage of 0.2 mg/kg IM q8h until discharge, which was 48 h after the procedure. 

After the chemical sterilization procedure, the dogs were submitted to a routine clinical evaluation once a day until discharge, as previously reported [18]. The parameters evaluated during clinical observation included body weight, rectal temperature, heart and respiratory rates, palpation of testis, scrotum, and inguinal region. An evaluation of firmness and tenderness by palpating the testicles was performed. The presence of inflammation (pain, redness, and heat) in the scrotal region was also detected. Further evaluations were performed at 15 and 30 days after discharge. 

None of the enrolled dogs showed side effects from the sclerosing agent, including signs of severe testicular inflammation or vocalization, abdominal muscle contraction or excessive movement from puncturing the scrotum during the injection. Furthermore, no side effects, such as vomiting or syncope, were observed using CEUS. No dogs showed marked inflammatory swelling of the testis 24 h after injection. Regarding palpation, only a slight increase in the firmness of testes was observed. Low pain scores were obtained after the procedure until clinical discharge. 

During CEUS, high-quality video clips were obtained, digitally stored on a hard disk, and subsequently analyzed by the same investigators (F.M. and S.D.). Post-processing quantitative analyses of the video clips were performed with image-analysis software (QontrastTM, Bracco, Milan, Italy) using parametric analysis of perfusion within a selected set of higher signal intensity frames in the region of interest (ROI). For each dog, one small oval ROI (area, 1.3 cm^2^) was drawn with the help of the software for normal testicular parenchyma before the CaCl_2_ solution was injected. After the treatment, another ROI of the same size was drawn close as possible to the injection site of the altered tissue. 

The image analysis software corrects translational movements in the ROI by selecting a gamma variate, bolus-corrected parametric curve model. For each previously determined ROI, time-intensity curves (TICs) were built. CEUS performed the tissue perfusion analysis based on video signal intensity (SI) changes over time. The maximum SI (100%) was defined as a white band in the greyscale bar (8-bit), assigning SI values to other pixels in the image based on this reference point. During the enhancement, the following parameters were automatically computed within the selected ROI: peak intensity (Peak), time-to-peak (TTP) measured from the injection time, mean transit time (MTT), regional blood volume (RBV), and regional blood flow (RBF). Peak intensity was defined as the percentage increase in SI—from 0 to a maximum intensity of 100—reached during the transit of the contrast agent at a specific time point. RBF was defined as the ratio between the RBV (proportional to the area under the curve) and MTT (circulation time of contrast agent in the investigated structure). Color-coded perfusion maps of RBFs were also performed. 

### 2.4. Statistical Analysis

Descriptive statistics were obtained for the perfusion variables tested. The obtained data, expressed as mean ± standard deviation (SD), were normally distributed (Kolmogorov-Smirnov test; *p* > 0.05). A paired Student’s t-test was applied to determine statistically significant effects of the experimental treatment. Values were considered significant at *p* < 0.05. Data were analyzed using the statistical software Prism v. 5.00 (GraphPad Software Ltd., San Diego, CA, USA, 2003).

## 3. Results

None of dogs included in the study showed clinical symptoms of disease; hematological and hematochemical parameters were within the reference range for the canine species [25,26].

Before the procedure, B-mode ultrasonography showed a normal aspect of the testicular parenchyma with a homogenous, medium-level, granular echotexture. When imaged in the longitudinal plane, the mediastinum testis appeared as an echogenic central band; this structure was seen as a small central focal echo in the transverse plane. After the intratesticular CaCl_2_ injection, conventional ultrasonography showed an anechoic echotexture of the testicular parenchyma that appeared heterogeneous with a less stippled appearance.

**Figure 1:**
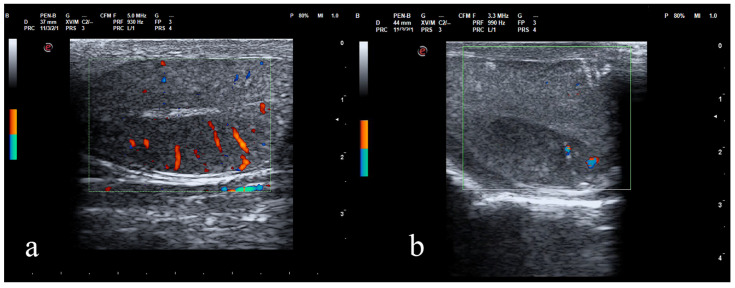
Doppler ultrasound of the dog testis before (**a**) and after (**b**) the intratesticular CaCl_2_ injection. Note the radial intraparenchymal vessels in pre-treatment testis (**a**) and the change of the vascular radial pattern in post-treatment testis (**b**).

The Color Doppler examination of pre-treatment testes detected a normal vascular signal with intraparenchymal vessels oriented in a radial pattern toward the mediastinum. After the treatment, the Doppler signal was visible, but the pattern had changed (Figure 1).

**Figure 2:**
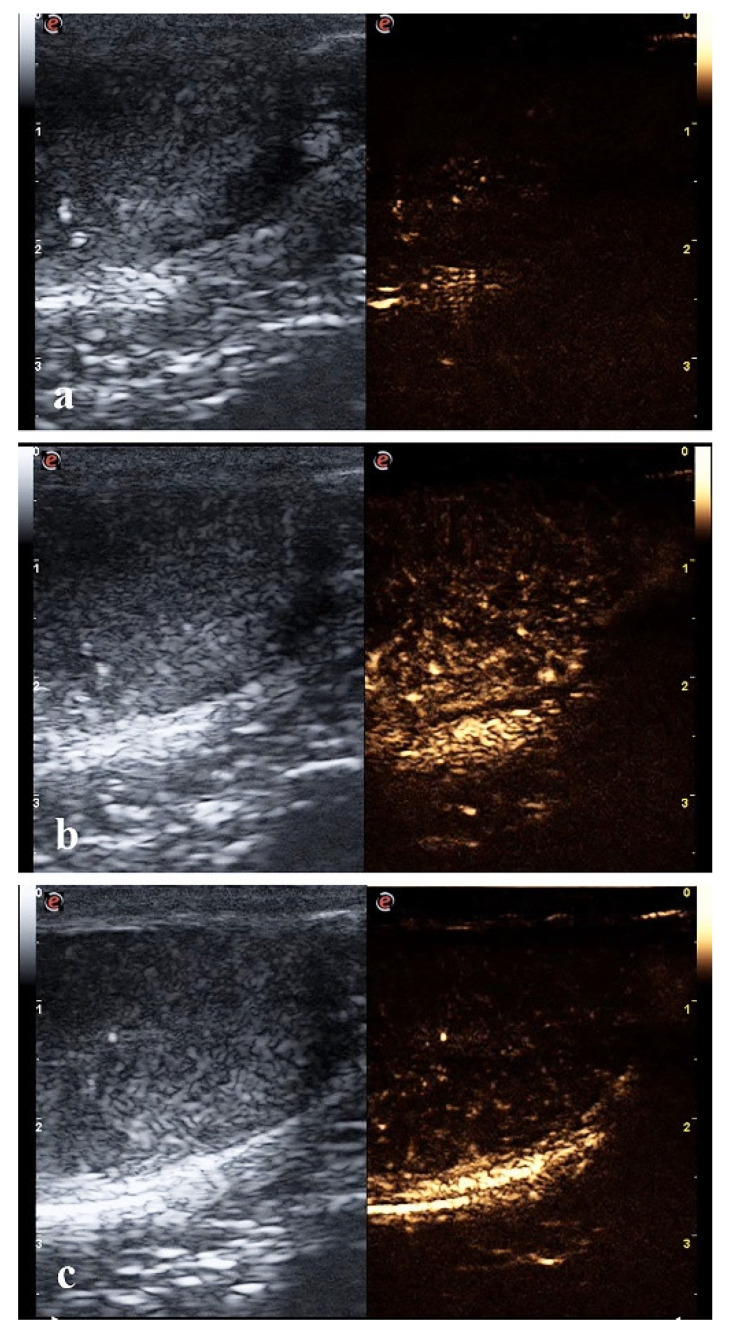
Contrast-enhanced ultrasound (CEUS) of the pre-treatment canine testis: images representing the perfusion pattern at the beginning of the wash-in phase at 11 s (**a**); peak at 20 s (**b**); and wash-out phase at 40 s (**c**).

Qualitative CEUS performed before the procedure showed a normal vascular pattern of the canine testes. Linear/striated macro- and microvascular flows outlined by microbubbles were detected. During the wash-in phase, which began about 11 s after the contrast injection, subcapsular arteries followed by intraparenchymal arteries showed a rapid and complete enhancement, with a homogeneous pattern of the testicular tissue at the peak intensity of 24.12 ± 6.4 s (Figure 2a,b). After the peak phase, an early wash-out began at about 24 s, characterized by a homogeneous decrease in tissue echogenicity (Figure 2c). This phase finished at about 45 s. 

**Figure 3:**
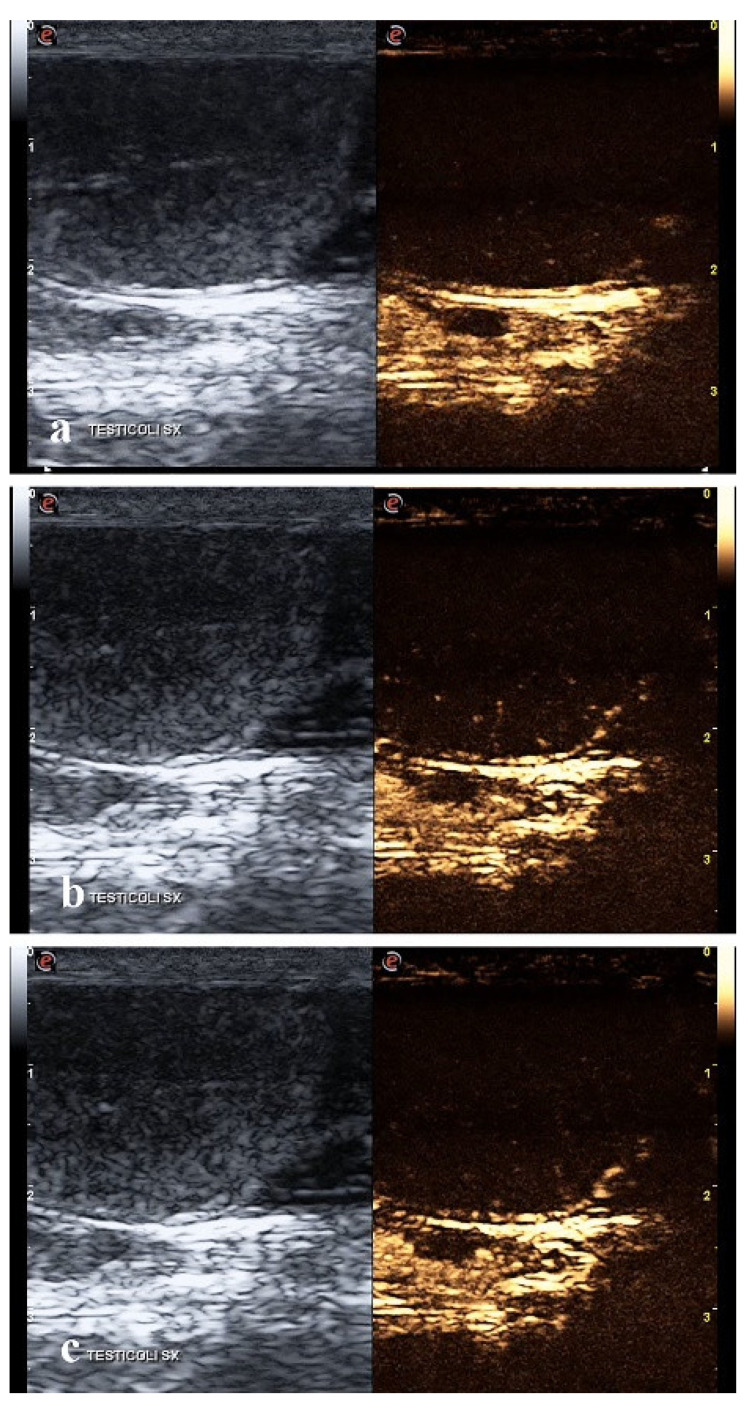
Contrast-enhanced ultrasound (CEUS) of the canine testis after CaCl_2_ injection: images representing the perfusion pattern at the beginning of the wash-in phase at 7 s (**a**); peak (**b**); and wash-out phase (**c**).

After the CaCl_2_ injection, qualitative CEUS showed an early wash-in phase that began about 7 s after the contrast injection (Figure 3a). At peak intensity, the parenchyma showed a lower intensity enhancement than pre-treatment testes, with a predominantly anechoic pattern and only a few hyperechoic vascular focal spots (Figure 3b). The wash-out phase occurred earlier than the healthy testis (Figure 3c).

**Figure 4:**
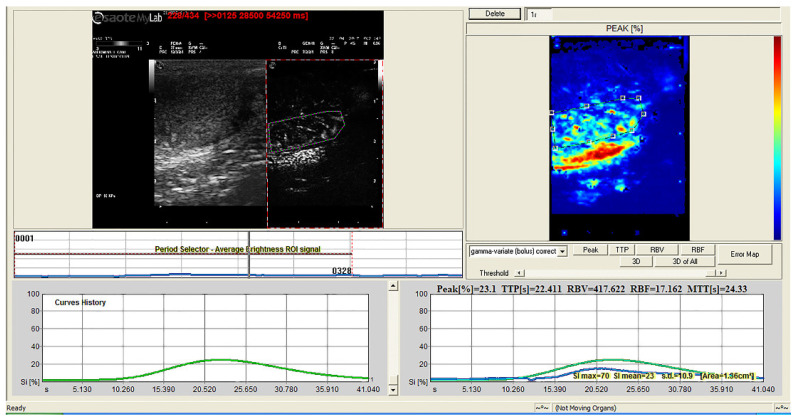
Quantitative analysis of contrast-enhanced ultrasound (CEUS): time–intensity curve of a normal canine testis created from a region of interest (ROI) positioned in the testicular parenchyma. Color-coded perfusion map of regional blood flow (**top right**).

**Figure 5:**
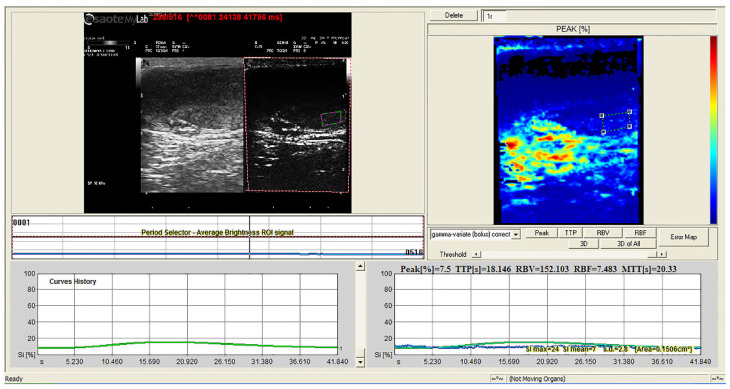
Quantitative analysis of contrast-enhanced ultrasound (CEUS): time–intensity curve of a canine testis after CaCl_2_ injection created from a region of interest (ROI) positioned in the testicular parenchyma. Color-coded perfusion map of regional blood flow (**top right**).

The quantitative CEUS showed different TIC parameters after treatment compared with before treatment. The treated dogs showed significantly lower signal intensity (Peak), blood volume (RBV), and weaker blood flow (RBF) (*p* < 0.001). The color-coded map of RBF also showed reduced tissue perfusion (Figure 4 and Figure 5). In Table 1, the mean values of each perfusion-related parameter before and after intratesticular CaCl_2_ injection with their statistical significance are reported.

## 4. Discussion

Nonsurgical chemo-sterilization by intratesticular injection of a sclerosing agent is a simple alternative to surgical castration in dogs. This method is less invasive with fewer risks, more economical than orchiectomy, and is suitable for mass-scale applications. Various chemical agents including zinc [2,10,11], glycerol [9], and calcium chloride [3,5,13,15,16] have been used for the same purpose, achieving varying outcomes. Several authors have explored chemical castration with CaCl_2_-associated solutions in different animals. In a previous study, we reported severe histological changes from a CaCl_2_ solution in the germinal epithelium of seminiferous tubules with large vacuolations and near-total disappearance of sperm cells [16]. In vitro studies on cultured endothelial cells demonstrated that CaCl_2_ effects on testicular vessels are likely caused by the activation of calcium signaling and nitric oxide pathways, followed by cell death with subsequently reduced blood perfusion and necrosis of the germinal epithelium. In vivo, sclerosants may also cause apoptosis in some tissues, leading to oxidative stress, inflammatory changes, and cellular degeneration [27].

Since no dogs in the present study showed altered body temperature, heart and respiratory rate, pain, or local signs of severe inflammatory reaction after the procedure, these findings underline that the intratesticular CaCl_2_ injection did not adversely affect physiological parameters and animal welfare, in agreement with previous studies [3,15,16,28]. 

In this study, we applied B-mode, Color Doppler, and CEUS to monitor vascular changes that may occur following the intratesticular injection of the CaCl_2_ sclerosing solution in enrolled dogs. 

Cost-efficient, safe, and clinically effective, CEUS has become a reference diagnostic imaging technique used in veterinary medicine to characterize prostatic and testicular diseases, assess perfusion kinetics, evaluate prostatic and testicular abnormalities, and distinguish between benign and malignant prostatic and testicular diseases [29,30,31,32,33,34,35,36].

Standard CEUS features of healthy canine testes have been reported in veterinary literature: CEUS enhances the vascular aspects of normal testicular parenchyma in about 15 s after the injection of the contrast agent into the bloodstream. The subcapsular arteries and marginal arteries show apparent enhancement during the wash-in phase. Then, the intratesticular arteries become enhanced. The contrast agent is gradually cleared from the parenchyma during the wash-out phase [37]. 

In a previous study, we reported greyscale and contrast ultrasonography features of the canine testicular parenchyma after the calcium chloride sterilant was injected, showing non-homogeneous parenchyma and reduced blood perfusion in the injected areas [3]. In the present study, qualitative and quantitative CEUS confirmed a significant reduction in blood perfusion and extended lesions in the canine testicular parenchyma after the sclerosing agent injection.

Statistical analysis proved that the peak intensity, regional blood volume, and regional blood flow were significantly lower in damaged than healthy testes. These changes may be due to the sclerosing action of CaCl_2_ in alcohol that causes a drastic reduction in testicular blood vessels, as highlighted by the decreased contrast agent concentration within the ROI. Time to peak and mean transit time showed no significant differences. This finding is probably due to reduced testicular perfusion localized to the injection site and does not affect the time required for the contrast agent to reach peak intensity in the testicular parenchyma or circulation time. 

In order to perform chemical sterilization, adequate anesthesia is required to block the transmission of pain and nociceptive signals and provide adequate analgesia [38]. In this study, the values of TIC perfusion parameters from pre-treatment healthy testes were higher than reported by other authors [25,39]. This discrepancy could be due to different sedatives used during the procedure that can affect the cardiac output, heart rate, and, consequently, the perfusion dynamic [40,41]. In this report, dogs were sedated using a 1:1 combination of tiletamine/zolazepam, affecting blood flow less than the medetomidine employed in the aforementioned study. 

In this study, we applied CEUS methodology to monitor vascular alterations due to the chemical sterilization of dogs. The sclerosing agent decreased vascularization of the parenchyma, which was reflected by CEUS sonographic findings. This diagnostic technique helps the clinician define immediate testicular injuries achieved by chemical castration. 

Our study presents some limitations. One of the main limitations was the small number of dogs enrolled. Further studies are needed to evaluate the safety and efficacy of this methodology on a larger number of subjects, including dogs under 16 weeks of age and animals with a broader weight range. Moreover, future studies can focus on the animal welfare implication of this method, including rate and postoperative complication. 

## 5. Conclusions

The results of our study demonstrate how injecting a CaCl_2_ solution into canine testicular parenchyma can be monitored by CEUS, which complements B-scale and Color Doppler ultrasound. The quantitative analysis of testicular perfusion determined by CEUS allowed us to assess the immediate severity of vascular injuries to testes after the procedure.

## Figures and Tables

**Table 1:** Statistical columns for each perfusion-related parameter in the testicular parenchyma before and after injecting the sclerosing agent, including their statistical significance.

		Min	Max	Mean ± SD	Lower 95% CI	Upper 95% CI	*p*-Value
TTP	Before	18.30	35.10	24.12 ± 6.43	16.13	32.11	0.48
	After	10.16	31.42	20.66 ± 8.16	10.53	30.80	
Peak	Before	18.50	24.70	21.60 ± 2.39	18.63	24.57	<0.0001
	After	2.60	7.50	4.72 ± 2.17	2.02	7.41	
RBV	Before	380.8	440.3	407.2 ± 22.62	379.1	435.3	0.0008
	After	51.71	195.0	134.3 ± 63.75	55.17	213.5	
RBF	Before	16.60	22.10	18.67 ± 2.19	15.95	21.39	<0.0001
	After	2.35	7.40	4.36 ± 2.18	1.64	7.08	
MTT	Before	20.20	27.50	22.84 ± 3.04	19.06	26.62	0.6411
	After	10.74	51.86	26.34 ± 17.36	4.78	47.89	

CI = Confidence Interval; Peak = peak enhancement; TTP = time to peak; RBV = red blood volume; RBF = red blood flow; MTT = mean transit time.

## Data Availability

The data presented in this study are available on request from the corresponding author.
